# Production of succinate with two CO_2_ fixation reactions from fatty acids in *Cupriavidus necator* H16

**DOI:** 10.1186/s12934-024-02470-6

**Published:** 2024-07-05

**Authors:** Linqing Li, Xiuyuan Zhou, Zhuoao Gao, Peng Xiong, Xiutao Liu

**Affiliations:** 1 School of Life Sciences and Medicine, Shandong University of Technology, Zibo, 255000 China; 2 International Joint Laboratory on Extremophilic Bacteria and Biological Synthesis, Shandong University of Technology, Zibo, 255000 China

**Keywords:** Carbon fixation, Succinate biosynthesis, 3HP cycle, *Cupriavidus necator* H16

## Abstract

**Background:**

Biotransformation of CO_2_ into high-value-added carbon-based products is a promising process for reducing greenhouse gas emissions. To realize the green transformation of CO_2_, we use fatty acids as carbon source to drive CO_2_ fixation to produce succinate through a portion of the 3-hydroxypropionate (3HP) cycle in *Cupriavidus necator* H16.

**Results:**

This work can achieve the production of a single succinate molecule from one acetyl-CoA molecule and two CO_2_ molecules. It was verified using an isotope labeling experiment utilizing NaH^13^CO_3_. This implies that 50% of the carbon atoms present in succinate are derived from CO_2_, resulting in a twofold increase in efficiency compared to prior methods of succinate biosynthesis that relied on the carboxylation of phosphoenolpyruvate or pyruvate. Meanwhile, using fatty acid as a carbon source has a higher theoretical yield than other feedstocks and also avoids carbon loss during acetyl-CoA and succinate production. To further optimize succinate production, different approaches including the optimization of ATP and NADPH supply, optimization of metabolic burden, and optimization of carbon sources were used. The resulting strain was capable of producing succinate to a level of 3.6 g/L, an increase of 159% from the starting strain.

**Conclusions:**

This investigation established a new method for the production of succinate by the implementation of two CO_2_ fixation reactions and demonstrated the feasibility of ATP, NADPH, and metabolic burden regulation strategies in biological carbon fixation.

**Supplementary Information:**

The online version contains supplementary material available at 10.1186/s12934-024-02470-6.

## Background

The “greenhouse effect” gradually intensifies as a huge number of greenhouse gas emissions are released. United Nations Secretary-General Antonio Guterres stated in July 2023 that “The era of global warming has ended; the era of global boiling has arrived.” It is noteworthy that the proportion of CO_2_ in global greenhouse gas has risen to 76.7% (v/v), making it the primary cause of climate change [1]. Consequently, reducing CO_2_ levels in the atmosphere has become a priority. In order to accomplish this objective, a viable approach involves the conversion of CO_2_ into valuable multi-carbon chemicals via the engineering of biological processes.

Over the course of the last four billion years, several complicated mechanisms for CO_2_ fixation have emerged in the natural world [2]. To present, a total of eight natural mechanisms for CO_2_ fixation have been confirmed. These mechanisms include the Calvin-Benson-Bassham (CBB) cycle, the 3-hydroxypropionate (3HP) cycle [3], the 3-hydroxypropionate-4-hydroxybutyrate (3HP/4HB) cycle [4], the reductive tricarboxylic acid (rTCA) cycle [5], the Wood-Ljungdahl (WL) pathway [6], the dicarboxylate/4-hydroxybutyrate (DC/4HB) cycle [7], the reductive glycine pathway [8], and the reverse oxidative TCA cycle [9]. Each carbon dioxide fixing mechanism has distinct properties that dictate its range of applicability. For example, the process of the WL pathway is limited to absolutely anaerobic settings because of the high sensitivity of the CO dehydrogenase/acetyl-CoA synthase to oxygen. The oxygen-sensitive enzymes in DC/4HB cycle and rTCA cycle, leading them just to operate under conditions of anaerobic and microaerobic. Liu et al. completed the task of compiling an extensive dataset encompassing various natural pathways for CO_2_ fixation. This dataset includes information on the sensitivity to oxygen, ATP requirement, thermodynamics, enzyme kinetics, and carbon species associated with these pathways, which indicate that the WL pathway and the 3HP cycle are the most appropriate mechanisms for anaerobic and aerobic CO_2_ fixation, respectively [2]. However, an ideal mechanism for one-carbon use in common synthetic biology platforms, would be capable of functioning in a robust manner under both aerobic and anaerobic conditions [10]. Therefore, this article selects the 3HP cycle as the research object, focusing on its application in the synthesis of high-value-added chemicals by fixing CO_2_.

**Fig. 1:**
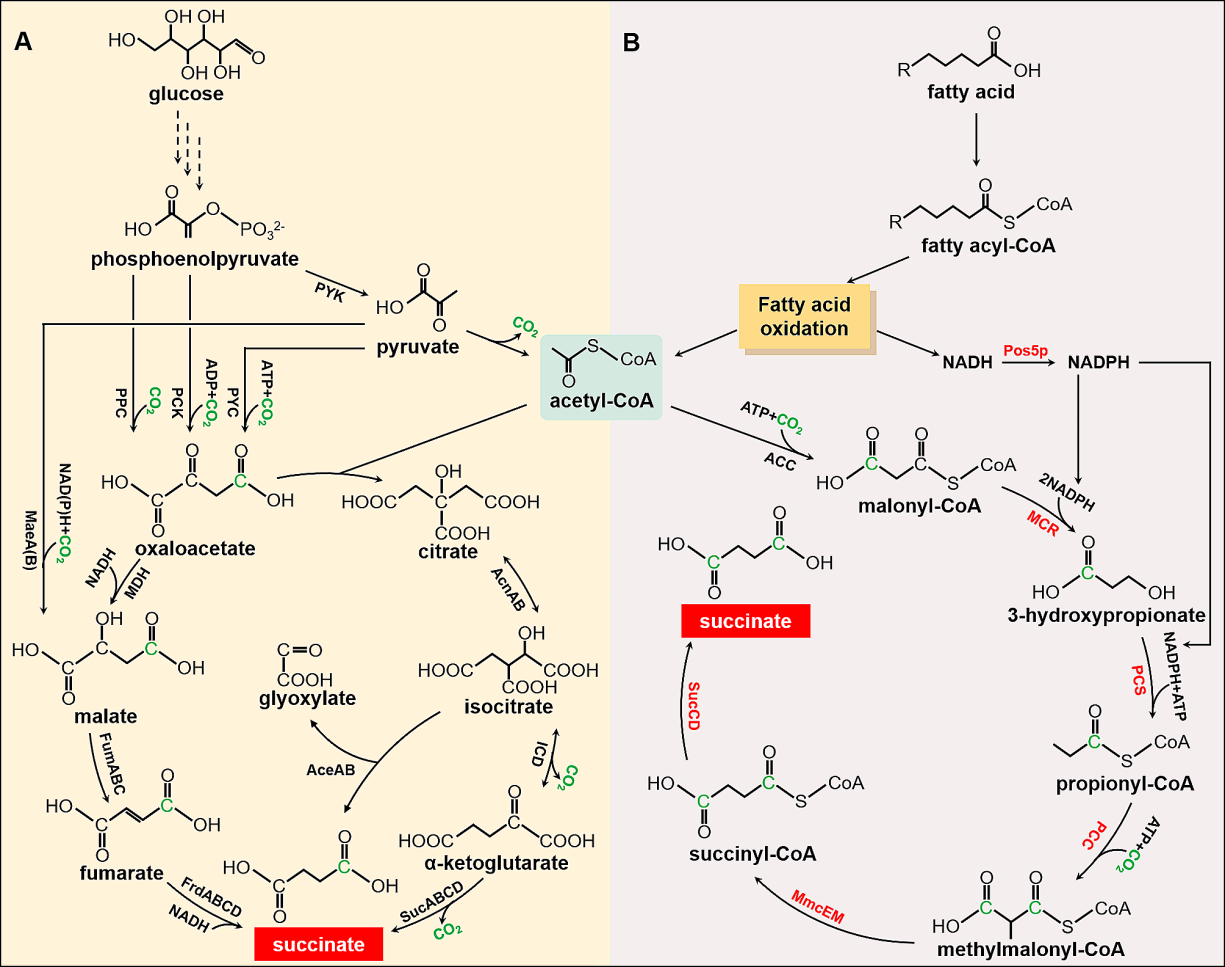
Succinate biosynthetic pathway. (**A**) Traditional succinate biosynthetic pathway with one CO_2_ fixation reaction. (**B**) Succinate biosynthetic pathway with two CO_2_ fixation reactions in this study. PCC, phosphoenolpyruvate carboxylase; PCK, phosphoenolpyruvate carboxykinase; PYC, pyruvate carboxylase; PYK, pyruvate kinase; ACC, acetyl-CoA carboxylase; MCR, malonylCoA reductase; PCC, propionyl-CoA carboxylase; PCS, propionyl-CoA synthase; MmcEM, methylmalonyl-CoA epimerase and mutase; SucCD, succinyl-CoA synthetase; MDH, malate dehydrogenase; FumABC, fumarate hydratase; FrdABCD, succinate dehydrogenase; AceAB, isocitrate lyase; The carbon atoms from CO_2_ were highlighted in green. The heterologous genes were highlighted in red

The 3HP cycle was discovered in photosynthetic green nonsulfur bacteria such as *Chloroflexus aurantiacus* [11]. Of the several natural carbon fixation processes, this cycle stands out as the most complicated, including a total of 16 enzymatic reaction steps that are catalyzed by a set of 13 enzymes. The 3HP cycle uses acetyl-CoA as the initial substrate and assimilates bicarbonate as carbon source by two CO_2_-fixing enzymes: acetyl-CoA carboxylase (ACC) and propionyl-CoA carboxylase (PCC) [12]. Up to now, a portion of the 3HP cycle has been added to *E. coli* in order to facilitate the conversion of CO_2_ into the synthesis of valuable compounds, including 3HP and succinate [13]. However, glucose was utilized as a source of carbon in these studies, and CO_2_ will be produced in the transformation of pyruvate into acetyl-CoA, which lead to loss of carbon and inefficient biosynthesis process (Fig. 1). Therefore, a carbon source utilization strategy with higher carbon yield is required. Here, we propose using fatty acids as the alternative biomass to drive CO_2_ fixation using a portion of 3HP cycle for the production of succinate in *Cupriavidus necator* H16.

Fatty acids can be derived from a range of cost-effective feedstocks, including waste cooking oil, sometimes referred to as “gutter oil” [14]. Fatty acids, which have highly reduced long aliphatic hydrocarbon chains, are primarily catabolized through the β-oxidation pathway, resulting in the formation of acetyl-CoA without carbon loss [15]. In addition, the process of β-oxidation of fatty acids produces a substantial quantity of reducing power, which may be utilized effectively in carbon fixation mechanisms. Nevertheless, the majority of microbial strains cannot effectively use fatty acids [16]. Our previous studies found that *Cupriavidus necator* H16 maybe a good choice. *Cupriavidus necator* H16 is a Gram-negative bacterium that has a rod-shaped morphology and is a facultative chemolithoautotrophic β-proteobacterium, found mostly in soil and freshwater [17]. Owing to its metabolic versatility, it has the capacity to use a wide spectrum of renewable resources. Throughout heterotrophic development, different carbohydrates, lipids and organic acids function as carbon and energy sources, whereas in the lack of organic substances, a mixture of H_2_, CO_2_, and O_2_ allows *Cupriavidus necator* H16 to grow autotrophically [18]. To the best of our knowledge, there have been few attempts of using fatty acids as carbon source to fix CO_2_ for succinate production in *Cupriavidus necator* H16.

Succinate, a dicarboxylic acid that is generated as an intermediary compound in the TCA cycle, has a wide range of uses as a foundational substance for the synthesis of diverse derivatives. In the conventional pathway of succinate biosynthesis, the generation of succinate is achieved by the carboxylation of phosphoenolpyruvate or pyruvate. This process leads to the fixation of one CO_2_ molecule and the subsequent creation of one molecule of succinate (Fig. 1A) [12, 19]. However, the CO_2_ fixation capacity for succinate biosynthesis can still be improved. We propose using a portion of the 3HP cycle to build a novel method for succinate production. Via two types of carbon fixing enzymes (ACC and PCC) in the 3HP cycle, each mol of succinate can be produced from one acetyl-CoA molecule and two molecules of CO_2_. The CO_2_ fixation capacity of this strategy is 2-fold higher than that of the conventional approach (Fig. 1B).

1 $$fatty\, acids \left(C16\right)+2\, ATP\to 8\, acetyl\, CoA+7\, NADH+7\, {FADH}_{2}$$

2 $$acetyl\, CoA+3\, ATP+3\, NADPH+2\, {CO}_{2}\to\, succinate+GTP$$

3 $$fatty\, acids \left(C16\right)\to 28.5\, ATP+31\, NADH+16\, {CO}_{2}$$

4 $$1\, {FADH}_{2}\to 1.5\, ATP$$

5 $$1.55\, fatty\, acids \left(C16\right)+7.23\, {CO}_{2}\to 8\, succinate+8.13\, ATP$$

When using fatty acids as the carbon source (taking palmitic acid as an example), the stoichiometry of acetyl-CoA production from fatty acids can be calculated by Eq. (1). Utilizing the 3HP bypass proposed in this study, the stoichiometry for converting acetyl-CoA and CO_2_ to succinate can be calculated by Eq. (2). According to Eq. (2), the carbon fixation process in this study requires additional reducing force and ATP. In addition, the complete oxidation process of fatty acids can be calculated by Eq. (3). The conversion between FADH_2_ and ATP can be calculated by Eq. (4). Thus, combining Eq. (1)+(2)+(3)+(4), the stoichiometry of succinate from CO_2_ and fatty acids via 3HP bypass can be calculated by Eq. (5). Using Eq. 5, we can calculate the theoretical yield of succinate is 2.37 g/g and the theoretical net fixation capacity of CO_2_ is 0.8 g/g via the 3HP bypass in this study. Comparatively, when calculating the theoretical yield using the original natural route of assimilating acetyl-CoA to produce succinate (the glyoxylate shunt), the theoretical yield of succinate is just 1.84 g/g, which is less than the theoretical yield of 2.37 g/g in this study. Meanwhile, if utilizing glucose as the carbon source, the 3HP bypass pathway would just demonstrate a theoretical succinate yield of 1.02 g/g, lower than the 2.37 g/g yield using fatty acids as the carbon source. Similarly, the theoretical net CO_2_ fixation capacity of 0.054 g/g via the 3HP bypass with glucose as the carbon source falls short of the 0.8 g/g capacity in this study (Fig S3). In conclusion, the production of succinate from fatty acids through a portion of the 3HP cycle in *Cupriavidus necator* H16 represents a new method with substantial advantages.

In addition, for a metabolic pathway with multiple enzymes, the production is affected by numerous factors including but not limited to functional balance between enzymes, enzyme activity, supply of precursors, energy and reducing power. Special attention must be paid to the supply of ATP and reducing power in this study, as two ATP molecules and three NADPH molecules are required for the production of one succinate molecule via this portion of 3HP cycle. In this study, the new pathway for succinate biosynthesis based on a portion of 3HP cycle was first constructed in *Cupriavidus necator* H16. Then the feasibility of this pathway was confirmed through isotope labeling validation experiments. At last, different approaches including the optimization of ATP and NADPH supply, optimization of metabolic burden, optimization of carbon sources were used to further optimize the fixation of CO_2_ and the biosynthesis of succinate.

## Methods

### Strains and chemicals

The *E. coli* strain DH5α was employed as the host strain for the production of the plasmids. *Cupriavidus necator* H16 was used as the host strain for CO_2_ fixation and succinate biosynthesis. The NaH^13^CO_3_ (99%) was acquired from Cambridge Isotope Laboratories (USA). Additional chemicals were obtained from Macklin (Shanghai, China) or Sinopharm (Beijing, China). Plasmid creation was conducted using the ClonExpress II One Step Cloning Kit, manufactured by Vazyme Biotech located in Nanjing, China. The PCR was performed employing PrimeSTAR Max DNA Polymerase, manufactured by Takara in Dalian, China. The strains and plasmids utilized during the study were illustrated in Table 1. All of primers used in this study were listed in Table S1. The growth of *E. coli* cells was facilitated employing LB medium, which consisted of 10 g/L tryptone, 5 g/L yeast extract, and 10 g/L NaCl. The cultivation of *Cupriavidus necator* H16 was performed utilizing a specific growth medium, namely SOB medium. This medium consisted of the following components: 20 g/L tryptone, 5 g/L yeast extract, 0.5 g/L NaCl, 2.5 mM KCl, 10 mM MgCl_2_, and 10 mM MgSO_4_. In addition, 14.04 g/L K_2_HPO_4_·3H_2_O, 5.24 g/L KH_2_PO_4_, 1 g/L NH_4_Cl, 1 g/L yeast extract, 0.5 g/L NaCl, 1 g/L MgSO_4_, 3 g/L NaHCO_3_, 50 mg/L biotin, 10 mg/L vitamin B12, 15 g/L fatty acids or oil were used for CO_2_ fixation and succinate production. Chloramphenicol (50 mg/L) and ampicillin (100 mg/L) were used where suitable.

**Table 1:** Strains and plasmids used in this study

Strains and plasmids	Description	Source
**Strains**		
*E. coli* DH5α	F^–^*ΔlacU169(Φ80 lacZ ΔM15) hsdR17 recA1 endA1 supE44 gyrA96 thi-1 relA1*	Invitrogen
*Cupriavidus necator* H16	H16 Wild-type, gentamicin resistant (Gen^r^)	DSM 428
SA02	*Cupriavidus necator* H16/ pBBR1MCS-1-*accBC*-*pcs*/pBBR1MCS-4-*mcrN*-*mcrC*-*pcc*-*mmcEM*-*sucCD*	This study
SA03	*Cupriavidus necator* H16/ pBBR1MCS-1-*accBC*-*pcs-adk* /pBBR1MCS-4-*mcrN*-*mcrC*-*pcc*-*mmcEM*-*sucCD*	This study
SA04	*Cupriavidus necator* H16/ pBBR1MCS-1-*accBC*-*pcs-vhb* /pBBR1MCS-4-*mcrN*-*mcrC*-*pcc*-*mmcEM*-*sucCD*	This study
SA05	*Cupriavidus necator* H16/ pBBR1MCS-1-*accBC*-*pcs-ptxD* /pBBR1MCS-4-*mcrN*-*mcrC*-*pcc*-*mmcEM*-*sucCD*	This study
SA09	*Cupriavidus necator* H16/ pBBR1MCS-1-*accBC*-*pcs-adk-pntAB*/pBBR1MCS-4-*mcrN*-*mcrC*-*pcc*-*mmcEM*-*sucCD*	This study
SA10	*Cupriavidus necator* H16/ pBBR1MCS-1-*accBC*-*pcs-adk-pos5P*/pBBR1MCS-4-*mcrN*-*mcrC*-*pcc*-*mmcEM*-*sucCD*	This study
SA11	*Cupriavidus necator* H16/ pBBR1MCS-1-*accBC*-*pcs-adk-YfjB*/pBBR1MCS-4-*mcrN*-*mcrC*-*pcc*-*mmcEM*-*sucCD*	This study
SA13	*Cupriavidus necator* H16/ pBBR1MCS-1-*accBC*-*pcs-adk-pos5P*/pBBR1MCS-4-*mcrN*-*mcrC*-*pcc*-*mmcEM*	This study
SA14	*Cupriavidus necator* H16/ pBBR1MCS-1-*accBC*-*pcs-adk-pos5P*/pBBR1MCS-4-*mcrN*-*mcrC*-*pcc*-*sucCD*	This study
SA15	*Cupriavidus necator* H16/ pBBR1MCS-1-*accBC*-*pcs-adk-pos5P*/pBBR1MCS-4-*mcrN*-*mcrC*-*pcc*	This study
**Plasmids**		
pBBR1MCS-1	rep_pBBR1_Cm^R^*lacI* P_lac_	Novagen
pBBR1MCS-4	rep_pBBR1_Amp^R^*lacI* P_lac_	Novagen
pBBR1MCS-1-*accBC*-*pcs*	rep_pBBR1_Cm^R^*lacI* P_lac_*accBC* P_lac_*pcs*	This study
pBBR1MCS-4-*mcrN*-*mcrC*-*pcc*-*mmcEM*-*sucCD*	rep_pBBR1_Amp^R^*lacI* P_lac_mcr_1−549_ P_lac_*mcr*_550−1219(N940V K1106W S1114R)_ P_lac_*pcc*_(N220I I391T)_ P_lac_*mmcEM*-*sucCD*	This study
pBBR1MCS-4-*mcrN*-*mcrC*-*pcc*-*mmcEM*	rep_pBBR1_Amp^R^*lacI* P_lac_mcr_1−549_ P_lac_*mcr*_550−1219(N940V K1106W S1114R)_ P_lac_*pcc*_(N220I I391T)_ P_lac_*mmcEM*	This study
pBBR1MCS-4-*mcrN*-*mcrC*-*pcc*-*sucCD*	rep_pBBR1_Amp^R^*lacI* P_lac_mcr_1−549_ P_lac_*mcr*_550−1219(N940V K1106W S1114R)_ P_lac_*pcc*_(N220I I391T)_ P_lac_*sucCD*	This study
pBBR1MCS-4-*mcrN*-*mcrC*-*pcc*	rep_pBBR1_Amp^R^*lacI* P_lac_mcr_1−549_ P_lac_*mcr*_550−1219(N940V K1106W S1114R)_ P_lac_*pcc*_(N220I I391T)_	This study
pBBR1MCS-1-*accBC*-*pcs-adk*	rep_pBBR1_Cm^R^*lacI* P_lac_*accBC* P_lac_*pcs* P_lac_*adk*	This study
pBBR1MCS-1-*accBC*-*pcs-vhb*	rep_pBBR1_Cm^R^*lacI* P_lac_*accBC* P_lac_*pcs* P_lac_*vhb*	This study
pBBR1MCS-1-*accBC*-*pcs-ptxD*	rep_pBBR1_Cm^R^*lacI* P_lac_*accBC* P_lac_*pcs* P_lac_*ptxD*	
pBBR1MCS-1-*accBC*-*pcs-adk-pntAB*	rep_pBBR1_Cm^R^*lacI* P_lac_*accBC* P_lac_*pcs* P_lac_*adk* P_lac_*pntAB*	This study
pBBR1MCS-1-*accBC*-*pcs-adk-pos5P*	rep_pBBR1_Cm^R^*lacI* P_lac_*accBC* P_lac_*pcs* P_lac_*adk* P_lac_*pos5P*	This study
pBBR1MCS-1-*accBC*-*pcs-adk-YfjB*	rep_pBBR1_Cm^R^*lacI* P_lac_*accBC* P_lac_*pcs* P_lac_*adk* P_lac_*YfjB*	This study

### Construction of plasmids

Molecular cloning was conducted employing typical protocols [20]. The genes encoding AccBC from *E. coli* (No. CAQ32718.1 and CAQ33581.1) and PCS from *Chloroflexus aurantacus* (No. AAL47820.2) were cloned into pBBR1MCS-1 vector between *Kpn*I and *Sac*I sites. The *accB* and *accC* genes were not optimized for codons during amplification, while the *pcs* gene was optimized for codons through BGI Beijing (China) during synthesis. The *accB* and *accC* genes share the same *lac* promoter. The *accB* gene used the RBS provided by the vector, while *accC* gene used the original RBS of the gene. The *pcs* gene used the *lac* promoter and RBS provided by the vector. The pBBR1MCS-1 vector carries a chloramphenicol resistance gene. The genes encoding MCR from *Chloroflexus aurantacus* (No. AAS20429.1), PCC from *Bacillus subtilis* (No. CAB14323.2), SucCD (No. CAQ31193.1 and CAQ31194.1) from *E. coli*, and MmcEM from *Chloroflexus aurantacus* (No. ACL23899.1 and ACM54095.1) were cloned into pBBR1MCS-4 vector between *Kpn*I and *Xba*I sites. The *pcc* gene used the *lac* promoter and RBS provided by the vector and it was optimized for codons through BGI during synthesis. The *mcr* gene was divided into *mcrN* and *mcrC. mcrN* encoded 1-549 amino acids of MCR protein and *mcrC* encoded 550–1219 amino acids of MCR protein. The *mcrC* gene used the *lac* promoter and the *mcrN* gene used the P_lac P2−51_ promoter. Both *mcrN* and *mcrC* were optimized for codons through BGI during synthesis. The *mmcEM* and *sucCD* genes share the same *lac* promoter. The *sucCD* genes were not optimized for codons during amplification, while the *mmcEM* genes were optimized for codons through BGI during synthesis. The pBBR1MCS-4 vector carries the ampicillin resistance gene. In addition, the *accB* gene was used to express the BCCP domain of propionyl-CoA carboxylase. The *accC* gene was used to express the BC domain of propionyl-CoA carboxylase. The *pcc* gene was used to express the CT domain of propionyl-CoA carboxylase. After the expression of the above three genes, a complete propionyl-CoA carboxylase will be formed [13]. The genes encoding ADK from *Bacillus licheniformis* (No. AOP13161.1), Vhb from *Vitreoscilla sp. C1* (No. AAA75506.1) or PtxD from *Stutzerimonas stutzeri* (No. QGZ31571.1) were cloned into pBBR1MCS-1 vector between *Sal*I and *Hind*III sites, respectively. The above gene was optimized for codons through BGI during synthesis. The genes encoding Pos5 from *Saccharomyces cerevisiae* (No. DAA11247.1), PntAB from *E. coli* (No. CAA0088984.1 and CAA0088981.1) or yfjB from *Bacillus subtilis* (No. AGG60168.1) were cloned into pBBR1MCS-1 vector between *BamH*I and *Spe*I sites, respectively. The above gene was optimized for codons through BGI during synthesis.

### Isotope labeling detection and succinate fermentation production

In order to confirm the practicability of CO_2_ fixation pathway, the engineered strain was cultured in 2 L fermentor and the products were detected by Mass spectrometry. The fermentor contained 1 L growth medium (14.04 g/L K_2_HPO_4_·3H_2_O, 5.24 g/L KH_2_PO_4_, 1 g/L MgSO_4,_ 1 g/L NH_4_Cl, 1 g/L yeast extract, 0.5 g/L NaCl, 20 g/L palmitic acid, pH 7.0). Throughout the procedure of fermentation, the temperature was controlled at 30 °C and the pH was effectively regulated at a value of 7.0 by means of automated supplementation of 10% (v/v) ammonia water, while the stirring rate was established at 400 rpm. Antifoam was employed to avoid frothing if required. Following a fermentation period of 6 h, the culture was supplemented with 100 µM IPTG, 3 g/L NaH^13^CO_3_, 50 mg/L biotin, and 10 mg/L vitamin B12. A solution containing 50 mg/L biotin and NaHCO_3_ was added as a supplement every 12 h and antibiotics were added every 24 h. The fermentation procedure was conducted for a duration of 48 h. In succinate fermentation production, NaH^13^CO_3_ was replaced by NaHCO_3_.

In the detection of isotope experimental results, the samples underwent an initial filtration process using a 0.22 μm syringe filter. The purification of succinate in the culture supernatant was conducted using a Waters 2545 Preparative HPLC system, which was equipped with an Xterra Prep RP18 (Ireland) column measuring 7.8 × 150 mm. The eluent utilized in this experiment was ultrapure water, which was delivered at a flow rate of 3 mL/min. In succinate analysis experiment, a method of direct infusion analysis in ESI-TOF-MS (Bruker, USA) was used. The detection process was in negative-ion mode. The dry heater temperature was maintained at 200℃ and the dry gas at 3.0 mL/min flow rate. The natural isotope distribution of succinate was determined utilizing the Scientific Instrument Services website (www.sisweb.com/mstools/isotope.htm). In the process of quantifying the level of succinate, the samples underwent filtration using a 0.22 μm syringe filter. The products present in the culture supernatant were then evaluated employing an Agilent 1200 Infinity series HPLC system, which was equipped with an Aminex HPX-87 H column measuring 300 × 7.8 mm and manufactured by Bio-Rad, located in Hercules, CA. The eluent utilized in this investigation was ultrapure water containing 5 mM H_2_SO_4_, which was delivered at a flow rate of 0.5 mL/min. The oven temperature was consistently kept at 55℃ [13]. Concentration of succinate was calculated based on standard curves.

## Results and discussion

### Construction of the 3HP bypass pathway in *Cupriavidus necator* H16

Through a portion of 3HP cycle, one molecule of acetyl-CoA can be converted into one molecule of succinate, accompanied by the fixation of two molecules of CO_2_. In this pathway, the β-oxidation of fatty acids in *Cupriavidus necator* H16 can generate acetyl-CoA without carbon loss. The first step in the process involves the carboxylation of Acetyl-CoA by the enzyme Acetyl-CoA carboxylase (ACC), resulting in the production of malonyl-CoA. Subsequently, malonyl-CoA is transformed into 3-hydroxypropionate by the action of malonyl-CoA reductase (MCR). Subsequently, the conversion of 3-hydroxypropionate into propionyl-CoA is facilitated by the enzyme propionyl-CoA synthase (PCS). The carboxylation of propionyl-CoA can be catalyzed by the enzyme propionyl-CoA carboxylase (PCC), resulting in the formation of (S)-methylmalonyl-CoA. Subsequently, the enzymatic reactions catalyzed by methylmalonyl-CoA epimerase (MmcE) and methylmalonyl-CoA mutase (MmcM) facilitate the conversion of (S)-methylmalonyl-CoA to succinyl-CoA. At last, the catalyzation of succinyl-CoA into the desired end product, succinate, is facilitated by the enzyme succinyl-CoA synthetase (SucCD) (Fig. 1B). Previous studies have found that MCR can be divided into two functional parts: MCR-N and MCR-C, and their combined catalytic activity is greater than that of wild-type MCR [21]. Therefore, this study also split MCR. MCR-N contains 1-549 amino acids of MCR and MCR-C contains 550–1219 amino acids of MCR. Using directed evolution and other methods, researchers found that the activity of MCR_550 − 1219(N940V K1106W S1114R)_ and PCC_(N220I I391T)_ mutants were higher than that of wild-type proteins [13, 22]. Therefore, in order to achieve the highest catalytic efficiency, this study also introduced the above-mentioned mutants in this portion of 3HP cycle. In general, we cloned related genes to establish a succinate biosynthetic route in *Cupriavidus necator* H16, which incorporates two CO_2_ fixation reactions, yielding strain SA02. The protein expression result of engineering bacteria can be found in Fig S1. The target proteins can all be expressed normally.

After the construction of the engineering strain was completed, we cultured it in a 2 L bioreactor for 48 h and measured the product concentration. We found that the engineering strain SA02 accumulated a certain concentration of succinate, which was higher than the concentration accumulated by the wild-type *C. necator* H16 (data not show). This suggests that the introduction of the 3HP bypass pathway may be the direct cause of the increase in succinate production. Therefore, in order to further verify whether the 3HP bypass pathway in *C. necator* H16 can function, we conducted isotope labeling experiments for further verification.

### Validation of succinate biosynthesis coupling two CO_2_ fixation reactions

**Fig. 2:**
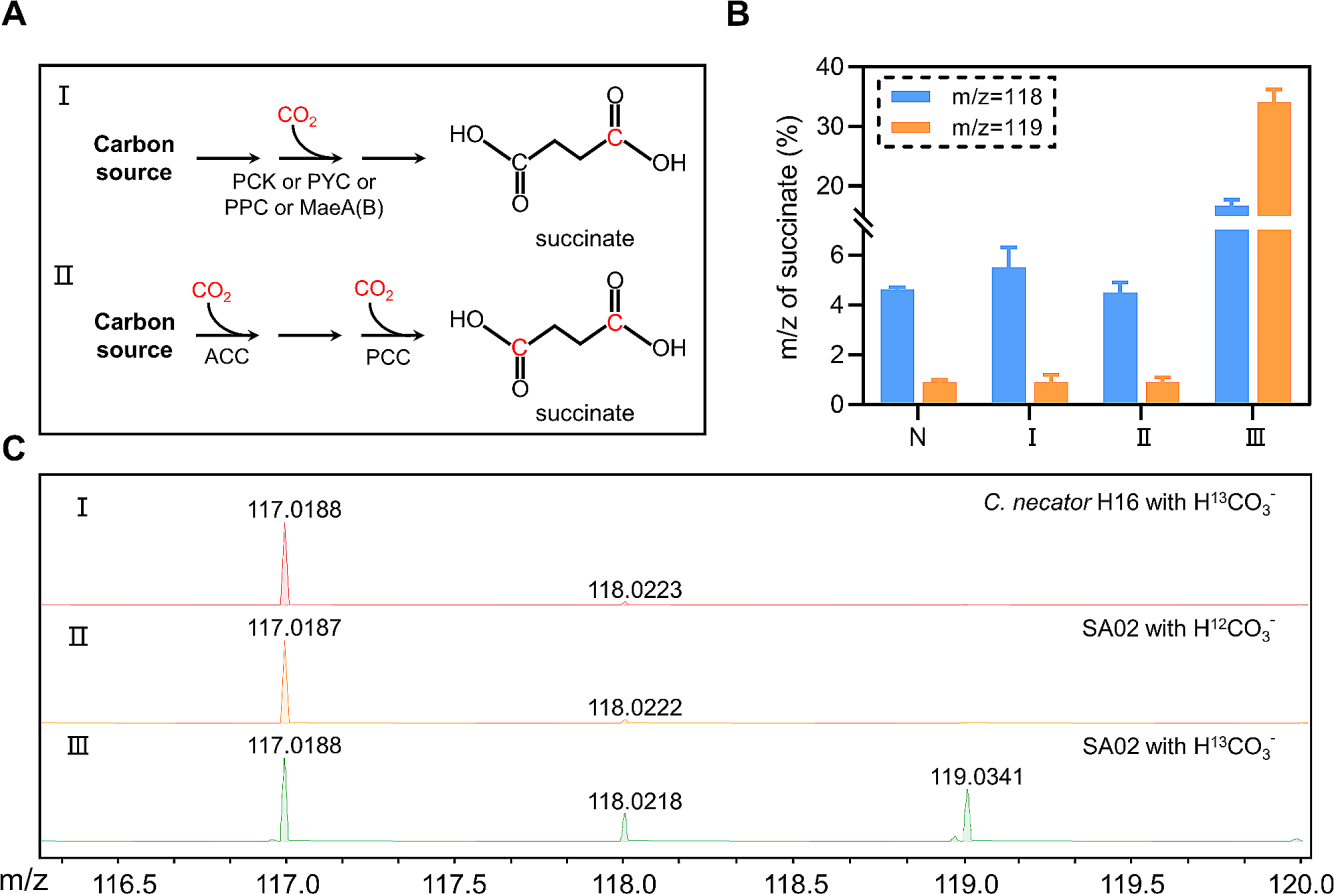
Biosynthetic pathway validation for succinate with two CO_2_ fixation reactions. (**A**) Schematic picture showing the carbon fixation capacity of different succinate biosynthetic pathways. (**B**) Percentage of succinate with different m/z produced by wild-type or engineered strains cultured with H^12^CO_3_^−^ or H^13^CO_3_^−^. “N” for the natural abundance of succinate with m/z of 118 and 119; “I” for wild-type strain cultured with H^13^CO_3_^−^; “II” for engineered strain cultured with H^12^CO_3_^−^; “III” for engineered strain cultured with H^13^CO_3_^−^; (**C**) Mass spectrum of succinate with different m/z produced by wild-type or engineered strains cultured with H^12^CO_3_^−^ or H^13^CO_3_^−^. SA02, engineering bacteria containing 3HP bypass pathway

Succinate is the key metabolite of the tricarboxylic acid (TCA) cycle, and it can also be created depending on PEP carboxylation, causing one CO_2_ molecule to be fixed and resulting in the production of one molecule of succinate. But in this work, two CO_2_ molecules can be fixed to synthesize one molecule of succinate (Figs. 1 and 2A and A). Thus, to differentiate whether the succinate is created through the heterologous mechanism we built, stable isotope labeling experiments were performed. The *Cupriavidus necator* H16 wild-type and SA02 strains were cultivated under aerobic conditions in a medium that was treated with either NaH^13^CO_3_ or NaH^12^CO_3_. Following the fermentation process, the succinate compound underwent purification by preparative HPLC and further analysis was conducted employing a mass spectrometer operating in the negative-ion mode. Succinate, a compound with a molecular weight of 118 g/mol, will have a m/z value of 117 in the negative-ion mode, assuming the absence of a ^13^C atom. The m/z value of 118 suggests the presence of a single ^13^C atom inside the succinate molecule. This incorporation is likely achieved by the process of PEP carboxylation during synthesis. The m/z value of 119 indicates the presence of two ^13^C atoms in the succinate molecule, indicating its synthesis via our specific mechanism. In the negative-ion mode, the natural abundance of succinate with m/z values of 118 and 119 was observed to be 4.6% and 0.9%, respectively. According to the findings presented in Fig. 2, it can be observed that when *Cupriavidus necator* H16 was provided with NaH^13^CO_3_, succinate with a m/z of 118 and 119 established approximately 5.5 ± 0.8% and 0.9 ± 0.3% respectively (Fig. 2B and C-I). These results suggest that, under the specified conditions, most of the succinate production by the *Cupriavidus necator* H16 strain occurs through the TCA cycle, while a smaller proportion is derived from PEP carboxylation. When the recombinant strain SA02 was supplemented with NaH^12^CO^3^, succinate distribution with different m/z was comparable to the natural abundance (Fig. 2B and C-II). When the SA02 strain was treated with NaH^13^CO_3_, there was a significant rise in the concentration of succinate with a m/z of 119, reaching 34.1 ± 2.1% (Fig. 2B and C-III). This finding provides evidence that the pathway implemented in this study is capable of synthesizing succinate from acetyl-CoA through two CO_2_ fixation reactions. In contrast to succinic acid generation by the carboxylation of PEP or pyruvate, this particular mechanism exhibited a greater CO_2_ fixation efficacy. In addition, the proportion of succinate with m/z of 118 has also shown an increase, reaching around 20%. This result is unexpected. It may suggest that due to the introduction of 3HP bypass, the metabolic flow distribution of engineering bacteria has changed, leading to the generation of some succinate through PEP or pyruvate carboxylation.

### Effect of ATP regulation strategy on succinate production

**Fig. 3:**
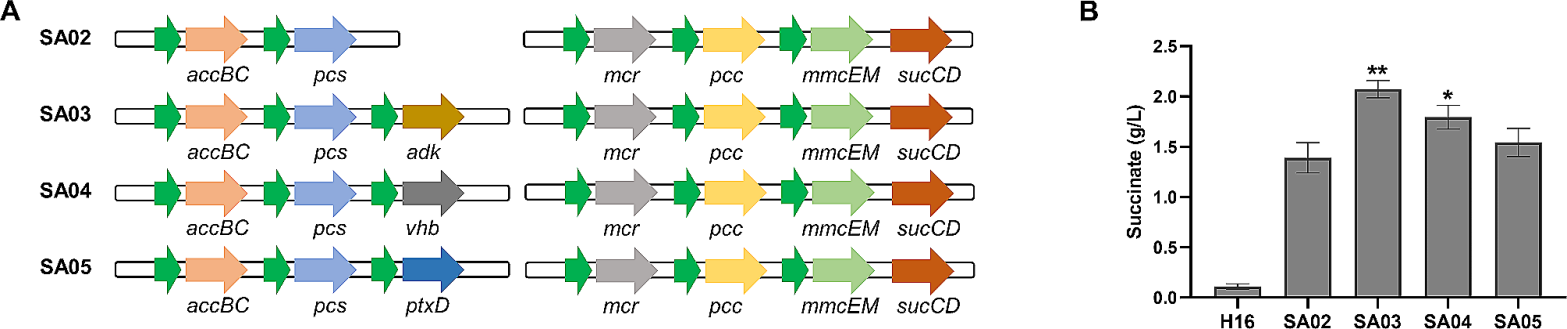
The ATP supply optimization for succinate production. (**A**) Schematic diagram of ATP-related gene expression plasmid. *adk*, the gene encoding adenylate kinase; *vhb*, the gene encoding *Vitreoscilla* hemoglobin; *ptxD*, the gene encoding phosphite dehydrogenase. (**B**) Effect of different ATP regulation strategies on succinate production. H16, wild type *Cupriavidus necator* H16; SA02, engineering bacteria containing 3HP bypass pathway; SA03, overexpression of *adk* gene in SA02 bacteria; SA04, overexpression of *vhb* gene in SA02 bacteria; SA05, overexpression of *ptxD* gene in SA02 bacteria. Data represent mean ± standard deviation (*n* = 3). *P < 0.05, **P < 0.01, vs. SA02

In the above carbon fixation pathway, one molecule of acetyl-CoA can be converted into one molecule of succinate with two molecules of CO_2_ fixation at the expense of two ATP molecules and three NADPH molecules. In addition, ATP functions as one of the most important driving forces, becoming a limiting factor for CO_2_ fixation. In this part, we adopted different ATP regulation strategies for enhancing succinate production, including regulating the O_2_ supply, optimizing the ADP supply, and altering NADH availability. Thus, different ATP regulation systems were constructed based on the introduction of *Vitreoscilla* hemoglobin, adenylate kinase, and phosphite dehydrogenase combined with the overexpression of the carbon fixation pathway. *Vitreoscilla* hemoglobin encoded by the *vhb* gene can bind O_2_ at a low extracellular O_2_ concentration, and supply it to the respiratory chain by direct interaction with the terminal respiratory cytochrome, which could facilitate O_2_ transfer and improve ATP supply [23]. The *ptxD* gene, which encodes phosphite dehydrogenase, is capable of catalyzing the oxidation of hydrogen phosphonate (phosphite) to phosphate in a nearly irreversible manner while reducing NAD^+^ to NADH. Intracellular NADH is the most important electron donor in the oxidative phosphorylation process. Enhancing NADH supply is also an efficient way to control intracellular ATP levels [24]. Furthermore, ADP is the direct substrate for ATP biosynthesis, and enhancing ADP supply is another effective way of increasing ATP production. AMP is the substrate for ADP synthesis, and adenylate kinase encoded by *adk* gene could catalyze the conversion of AMP to ADP [25]. *Adk*, *vhb*, and *ptxD* genes were transformed into SA02 strain to generate strains SA03, SA04, and SA05, respectively. It should be noted that, for the cultivation of strain SA05, 15 mM phosphite was added to the medium. As shown in Fig. 3, in conditions of aerobic fed-batch in 2 L bioreactor, each ATP regulation approach effected a rise of succinate production in diverse degrees. Among them, SA03 strain presented the maximum succinate production of 2.07 g/L, representing a 48.9% increase compared to SA02 strain (*P* < 0.01). It demonstrated that ADK is a good choice for enhancing succinate biosynthesis from ATP regulation strategy.

### Effect of NADPH regulation strategy on succinate production

**Fig. 4:**
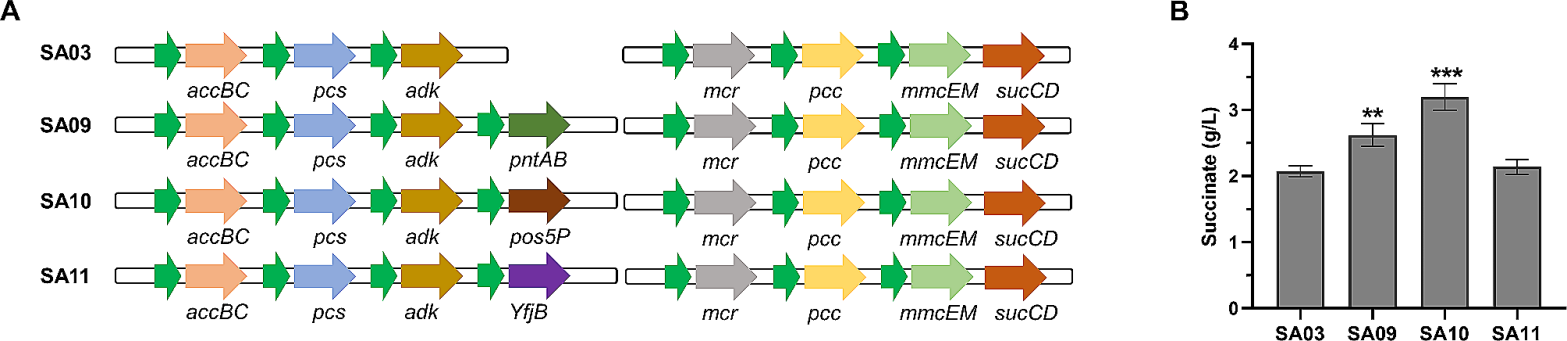
The NADPH supply optimization for succinate production. (**A**) Schematic diagram of NADPH-related gene expression plasmid. *pntAB*, the gene encoding NADP^+^ transhydrogenase; *pos5p*, the gene encoding NADH kinases; *yfjB*, the gene encoding NAD^+^ kinase. (**B**) Effect of different NADPH regulation strategies on succinate production. SA03, overexpression of *adk* gene in SA02 bacteria; SA09, overexpression of *pntAB* gene in SA03 bacteria; SA10, overexpression of *pos5p* gene in SA03 bacteria; SA11, overexpression of *yfjB* gene in SA03 bacteria. Data represent mean ± standard deviation (*n* = 3). **P < 0.01, ***P < 0.001, vs. SA03

Except for ATP, NADPH is another key factor for succinate production in this work. During fatty acid beta-oxidation, acetyl-CoA is produced as the direct substrate for succinate biosynthesis and NAD^+^ is metabolized to NADH at the same time [14, 18]. Consequently, how to direct the phosphorylation of NADH to NADPH is a potential way to increase NADPH regeneration [14]. In this part, we adopted three different NADPH regulation strategies for enhancing succinate production, including NADP^+^ transhydrogenase (PntAB; EC 1.6.1.2), NADH kinases (Pos5p; EC 2.7.1.86), and NAD^+^ kinase (YfjB; EC 2.7.1.23). Three enzymes use different ways for NADPH regeneration. PntAB can catalyze H^+^ transfer from NADH to NADP^+^, producing NADPH. Pos5p has a high affinity for NADH and could catalyze NADH phosphorylation to produce NADPH. YfjB could catalyze ATP-dependent phosphorylation of NAD^+^ to produce NADP^+^, which is the direct substrate of NADPH. PntAB, Pos5p, and YfjB genes were transformed into SA03 strain to generate strains SA09, SA10, and SA11, respectively. As shown in Fig. 4, in conditions of aerobic fed-batch in a 2 L bioreactor, each NADPH regulation strategy affected a rise in succinate creation to a different degree. Among them, the SA10 strain exhibited the most succinate production, reaching a concentration of 3.2 g/L. This is a notable 54.6% rise when compared to the succinate production of the SA03 strain (*P* < 0.001). These outcomes verified the significance of NADPH supply and demonstrated that Pos5p is a good choice for succinate production.

### Effect of metabolic burden and carbon sources on succinate production

**Fig. 5:**
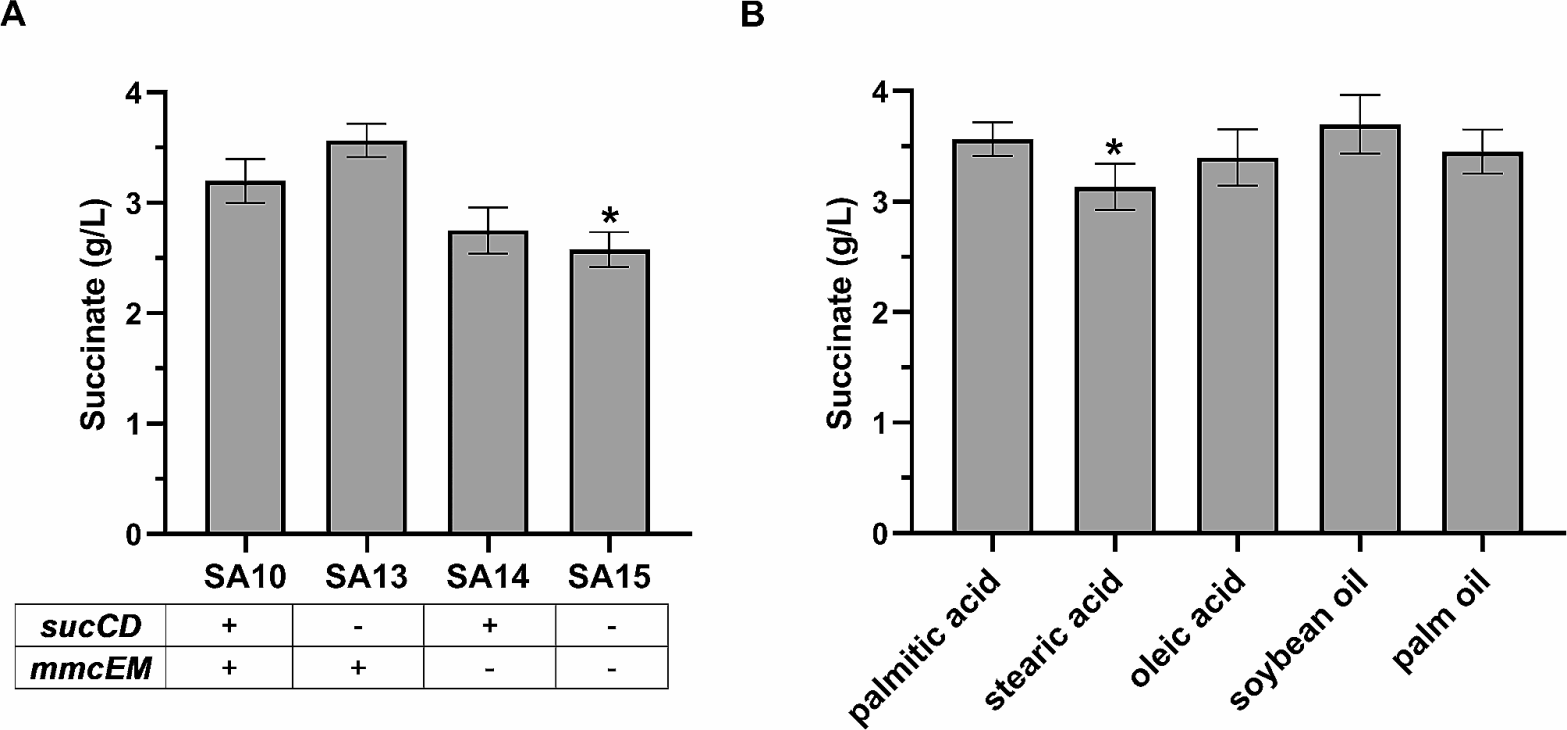
Effect of metabolic burden and carbon sources on succinate production. (**A**) Effect of metabolic burden on succinate production. SA13, lack of *sucCD* gene in SA10; SA14, lack of *mmcEM* gene in SA10; SA15, lack of *sucCD* and *mmcEM* genes in SA10. (**B**) Effect of carbon sources on succinate production of SA13 strain. Data represent mean ± standard deviation (*n* = 3). *P < 0.05 vs. control group

The biosynthesis of succinate with two carbon fixation reactions in this study involves multiple enzymes. Overexpression of multiple enzymes can increase the metabolic burden of engineering bacteria. Metabolic burden refers to the protein cost of expressing recombinant enzymes and it will negatively affect the efficiency of biosynthesis. One strategy to reduce metabolic burden is to replace overexpressed exogenous genes with endogenous genes. Due to the similarity in function of endogenous genes with *mmcEM* or *sucCD*, attempts were made to replace these two overexpressed genes with endogenous genes to reduce the host’s metabolic burden. We tested the individual omission of each gene and the omission of both. As shown in Fig. 5A, under aerobic fed-batch conditions in a 2 L bioreactor, reducing metabolic burden has a positive impact on increasing succinate production. Among them, SA13 strain which not overexpressed *sucCD* presented the highest succinate production of 3.6 g/L, an increase of 12.5% from the SA10 strain and 159% from the starting SA02 strain. It means managing the metabolic burden is useful for product biosynthesis.

Next, we evaluated the effects of yeast extract presented in the medium on succinate production. We measured the produced succinate from cultivation with SA13 strain grown on 1 g/L yeast extract supplemented media but without any fatty acids. As shown in Fig S2, the succinate production in the control group was only 0.2 g/L, which is only about 5.6% of the experimental group with added fatty acids. This indicates that when fatty acids were added to the culture medium, the vast majority of succinate (more than 94%) was obtained through the conversion of fatty acids. We also evaluated the effects of different carbon sources on succinate production of the SA13 strain. Palmitic acid, stearic acid, oleic acid, soybean oil, and palm oil were used as raw materials for the production of succinate at 2 L bioreactors. In order to make better use of soybean oil and palm oil, 20 mg/L lipase was added every 12 h during the fermentation. When using different carbon sources, the yield of succinate does not differ significantly, with soybean oil being the carbon source that slightly increases the yield (Fig. 5B). These results indicated the possibility of producing succinate using different fatty acids as carbon sources, and these raw materials (especially cheap oils) provide competitiveness for the low-cost biosynthesis of succinate with two carbon fixation reactions.

This new succinate pathway has made some progress but still has some unresolved issues. As mentioned, only 3.6 g/L succinate was produced with a yield of 0.24 g/g fatty acid at a 2 L bioreactor. Compared with the theoretical yield of 2.37 g/g fatty acid, the actual yield is only 10.12% of the theoretical yield, and there is still a lot of room for improvement. Possible reasons for the low yield are multiple, such as the presence of rate-limiting steps in metabolic pathways, difficulties in substrate utilization, and imbalanced enzyme expression in this multi-enzyme pathway. Therefore, some optimization strategies can be used in the future to further improve the yield. For example, we can choose to conduct metabolomics analysis and metabolic flux analysis. This can help us determine the rate-limiting steps of this pathway and understand the metabolic flux of 3HP bypass and other acetyl-CoA assimilation routes. It can also help us understand the bottlenecks in the synthesis process of intracellular cofactors, such as ATP or NADPH. It will provide guidance for further optimization of engineering strains. Furthermore, fatty acids, serving as carbon sources, have low solubility in fermentation broth. It will seriously affect the utilization of fatty acids and affect the production of succinate. It also makes it difficult to measure the biomass accumulation data in this work. One solution is to add surfactants to the fermentation medium to further promote the utilization of fatty acids and use the plate counting method to calculate biomass accumulation data. In addition, this work involves multiple proteins. From the protein expression results of engineering bacteria (figure S1), the protein expression is imbalanced. The imbalance of enzyme expression levels can also affect the synthesis efficiency of products [21]. Some solutions include RBS engineering or replacing the promoter. Except for the methods introduced in this work, there are also some other promising solutions to improve the supply of ATP and NADPH, such as powering bacteria or optimizing metabolic pathways [26].

## Conclusion

This study constructed and optimized a succinate biosynthetic pathway with double CO_2_ fixation reactions in recombinant *C. necator* H16 utilizing fatty acids as carbon sources. This work possesses advantages in several aspects: (1) This pathway has high carbon fixation efficiency. One molecule of succinate is produced from a single acetyl-CoA molecule and two CO_2_ molecules. It means that 50% of the carbon atoms in succinate are generated from CO_2_, which is twice that of the traditional succinate biosynthesis, depending on the PEP or pyruvate carboxylation. (2) Using fatty acid as raw material avoids carbon loss during acetyl-CoA and succinate production. Previous work used glucose as a carbon source, leading to CO_2_ release in the reaction of pyruvate to acetyl-CoA (the precursor of succinate in this pathway) [13]. This study uses fatty acids as raw material, which not only does not cause carbon loss in its β-oxidation, but also has a high yield of acetyl-CoA (for example, one molecule of palmitic acid can produce 8 molecules of acetyl-CoA). (3) Developed effective ATP and NADPH regulation strategies. Through the screening, this study proved ADK and Pos5p were good choices for ATP and NADPH dependent pathway in *C. necator* H16. Meanwhile, it also provides the solution for the utilization of waste oils.

### Electronic supplementary material

Below is the link to the electronic supplementary material.

Supplementary Material 1

## Data Availability

No datasets were generated or analysed during the current study.

## Abbreviations

ACC: Acetyl-CoA carboxylase
PCC: Propionyl-CoA carboxylase
3HP: 3-hydroxypropionate
