# Supplementary material for: Production of succinate with two CO2 fixation reactions from fatty acids in Cupriavidus necator H16
Source: Microb Cell Fact. 2024 Jul 5;23:194. doi: 10.1186/s12934-024-02470-6 (PMC11225152; doi:10.1186/s12934-024-02470-6)
Supplement: Supplementary file 1 — Supplementary Material 1 [file 12934_2024_2470_MOESM1_ESM.docx]

Table S1 Primers used in this study

| **Primer** | **Sequence** | **Restriction site** |
| --- | --- | --- |
| pBBR1MCS-4-*mcrN*-*mcrC*-*pcc*-*mmcEM*-*sucCD* construction | |  |
| *mcrN-mcrC* cloning | |  |
| *mcr*-F | cgataagcttgatatcgaattcactttacactttaagcttcatatg | EcoRⅠ |
| *mcr*-R | atcccccgggctgcaggaattcttacacggtaatcgcccgtccgcga | EcoRⅠ |
| *pcc* cloning | |  |
| *pcc*-F | cagcccgggggatccactagttaatgtgagttagctcactcattag | SpeⅠ |
| *pcc*-R | cgcggtggcggccgctctagattacagcggaatattgccatgtttct | XbaⅠ |
| *mmcEM* cloning | |  |
| *mmcEM*-F | agggaacaaaagctgggtaccgatgttcaccgctgttgaccacatc | KpnⅠ |
| *mmcEM*-R | gggggggccggtaccttaagcagcaacacctttcagacgt |  |
| *sucCD* cloning | | |
| *sucCD*-F | cggccccccctcgaggtcgacgatgaacttacatgaatatcaggc | SalⅠ |
| *sucCD*-R | aagcttatcgataccttatttcagaacagttttcagtgct |  |
| pBBR1MCS-1-*accBC*-*pcs* construction | |  |
| *accBC* cloning | |  |
| *accBC*-F | agggaacaaaagctgggtaccgatggatattcgtaagattaaaaaact | KpnⅠ |
| *accBC*-R | agggggggccggtaccttatttttcctgaagaccgagttttt |  |
| *pcs* cloning | |  |
| *pcs*-F | gccgccaccgcggtggagctctaatgtgagttagctcactcattag | SacⅠ |
| *pcs*-R | ggcgaattggagctcctaccgctcgccggccgtccacgcct |  |
| pBBR1MCS-1-*accBC*-*pcs-adk* construction | |  |
| *adk*-F | cccctcgaggtcgacgatgaacttagtcttaatgggactg |  |
| *adk*-R | ttcgatatcaagctttcatttgtcccggcctccgagaagctcc |  |
| pBBR1MCS-1-*accBC*-*pcs-ptxD* construction | |  |
| *ptxD*-F | cccctcgaggtcgacgatgctgccgaaactggtgattacccat |  |
| *ptxD*-R | ttcgatatcaagcttttagcacgccgccggttccgcctgc |  |
| pBBR1MCS-1-*accBC*-*pcs-vhb* construction | |  |
| *vhb*-F | cccctcgaggtcgacgatgctggatcagcagaccattaac |  |
| *vhb*-R | ttcgatatcaagcttttattccaccgcctgcgcatacagatcc |  |
| pBBR1MCS-1-*accBC*-*pcs-adk-pos5P* construction | |  |
| *pos5P*-F | ttcctgcagcccgggggatccatgtttgtcagggttaaattgaataaa | BamHⅠ |
| *pos5P*-R | cgctctagaactagtttaatcattatcagtctgtctcttgg |  |
| pBBR1MCS-1-*accBC*-*pcs-adk-pntAB* construction | |  |
| *pntAB*-F | ttcctgcagcccgggggatccatgcgcattggcattccgcgcgaac | BamHⅠ |
| *pntAB*-R | cgctctagaactagtttagtttttgcgaaacattttcag |  |
| pBBR1MCS-1-*accBC*-*pcs-adk-yfjB* construction | |  |
| *yfjB*-F | ttcctgcagcccgggggatccatgagcggcaacattgcggtgga | BamHⅠ |
| *yfjB*-R | cgctctagaactagtttagccaaagctaaatttgcctttca |  |


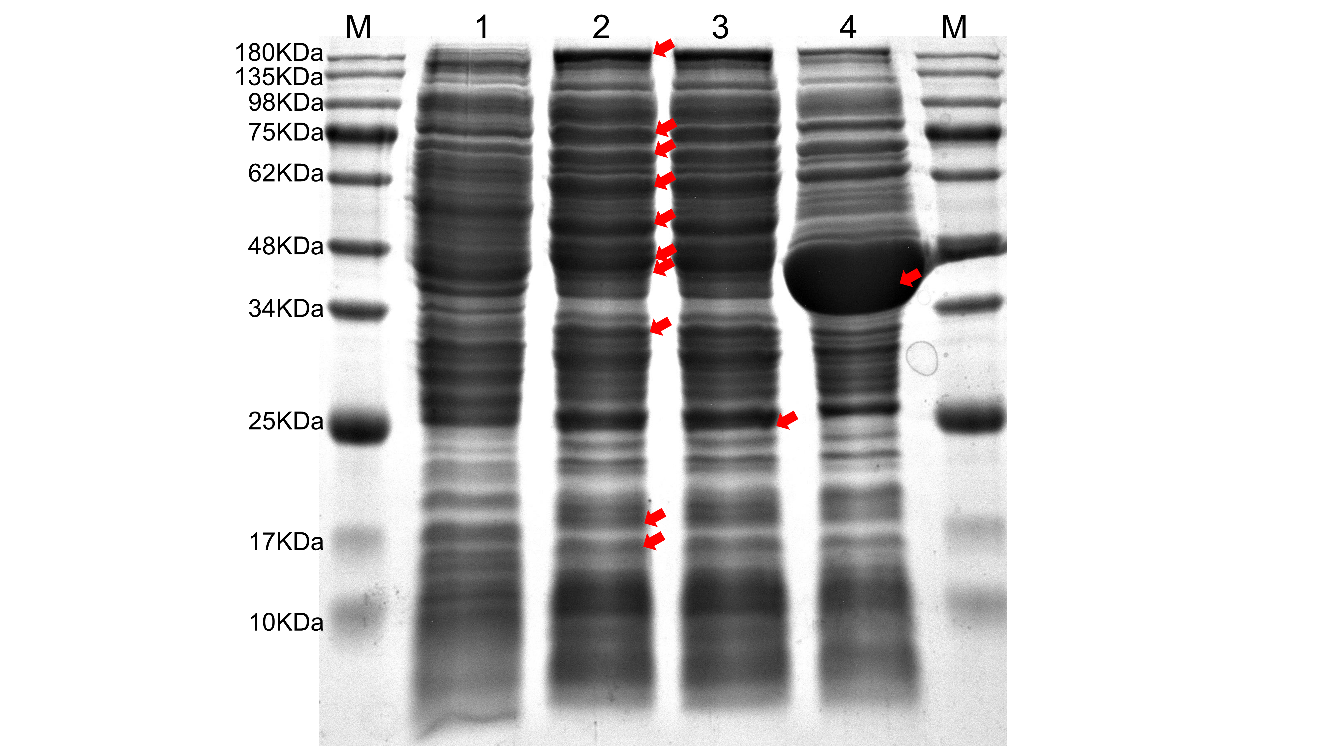


Figure S1: Coomassie brilliant blue-stained SDS-PAGE analysis of recombinant proteins from different constructs. Lane M, protein molecular weight marker. Lane 1, crude cell extracts from *Cupriavidus necator* H16. Lane 2, crude cell extracts from strain SA02. The positions corresponding to the overexpressed genes were indicated by an arrow. From top to bottom, there were PCS, MmcM, McrC, McrN, PCC, AccC, SucC, SucD, MmcE, AccB. Lane 3, crude cell extracts from SA03. The position corresponding to the *adk* gene was indicated by an arrow. Lane 4, crude cell extracts from SA10. The position corresponding to the *pos5P* gene was indicated by an arrow.


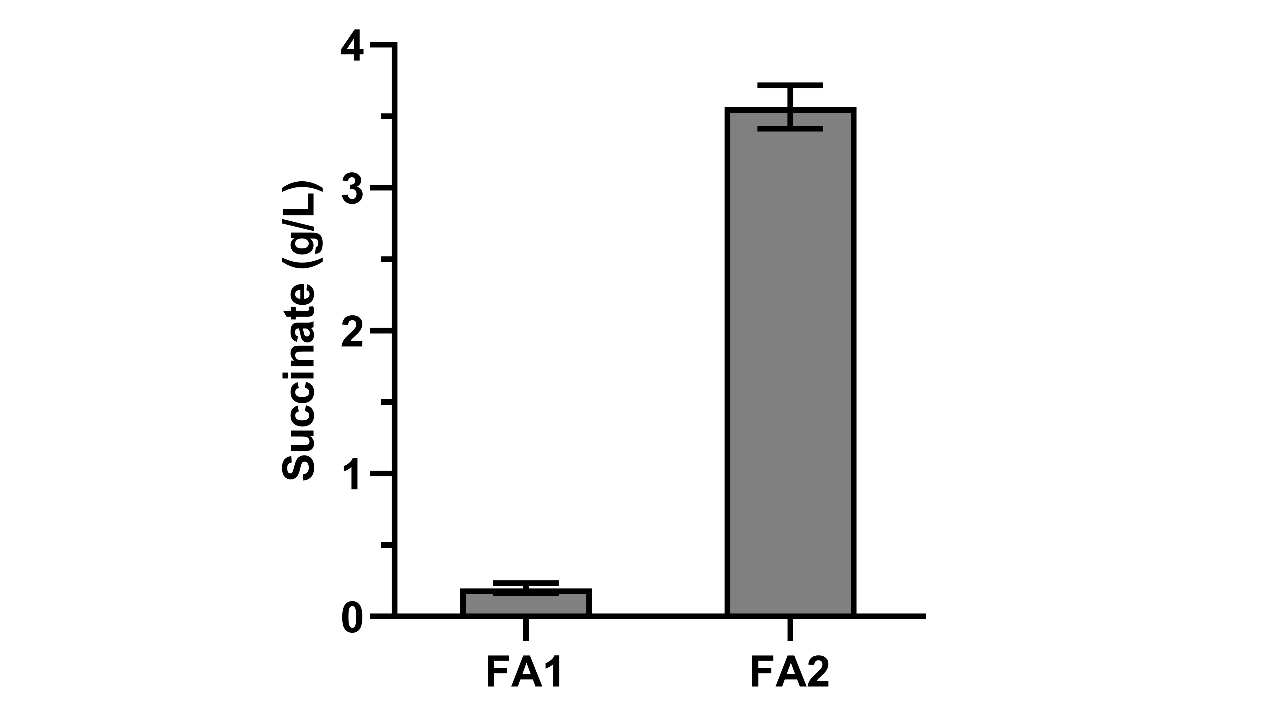
 Figure S2: Verify the contribution of yeast extract to succinate production. FA1, SA13 strain grown on 1g/L yeast extract supplemented media but without any fatty acids in the media. FA2, SA13 strain grown on 1g/L yeast extract supplemented media and with 15 g/L fatty acids in the media.

Figure S3: The stoichiometric equations involved in this study. When using fatty acids as the carbon source (taking palmitic acid as an example), the stoichiometry of acetyl-CoA production from fatty acids can be calculated by equation (1). Utilizing the 3HP bypass proposed in this study, the stoichiometry for converting acetyl-CoA and CO_2_ to succinate can be calculated by equation (2). The complete oxidation process of fatty acids can be calculated by equation (3). The conversion between FADH_2_ and ATP can be calculated by equation (4). Thus, combining equations (1)+(2)+(3)+(4), the stoichiometry of succinate from CO_2_ and fatty acids via 3HP bypass can be calculated by equation (5). The stoichiometry of succinate production from acetyl-CoA using the original natural route (the glyoxylate shunt) can be calculated by equation (6). Thus, combining equations (1)+(4)+(6), the stoichiometry of succinate from CO_2_ and fatty acids via the glyoxylate shunt can be calculated by equation (7). The complete oxidation process of glucose can be calculated by equation (8). The stoichiometry of acetyl-CoA production from glucose can be calculated by equation (9). Thus, combining equations (2)+(4)+(8)+(9), the stoichiometry of succinate from CO_2_ and glucose via the 3HP bypass can be calculated by equation (10)
